# Emerging Trends in Nanomedicine: Carbon-Based Nanomaterials for Healthcare

**DOI:** 10.3390/nano14131085

**Published:** 2024-06-25

**Authors:** Nargish Parvin, Vineet Kumar, Sang Woo Joo, Tapas Kumar Mandal

**Affiliations:** School of Mechanical Engineering, Yeungnam University, Gyeongsan 38541, Republic of Korea; nargish.parvin@gmail.com (N.P.); vineetfri@gmail.com (V.K.)

**Keywords:** carbon-based nanomaterials, carbon quantum dots (CQDs), carbon 2D nanosheets, biomedical applications, theranostics

## Abstract

Carbon-based nanomaterials, such as carbon quantum dots (CQDs) and carbon 2D nanosheets (graphene, graphene oxide, and graphdiyne), have shown remarkable potential in various biological applications. CQDs offer tunable photoluminescence and excellent biocompatibility, making them suitable for bioimaging, drug delivery, biosensing, and photodynamic therapy. Additionally, CQDs’ unique properties enable bioimaging-guided therapy and targeted imaging of biomolecules. On the other hand, carbon 2D nanosheets exhibit exceptional physicochemical attributes, with graphene excelling in biosensing and bioimaging, also in drug delivery and antimicrobial applications, and graphdiyne in tissue engineering. Their properties, such as tunable porosity and high surface area, contribute to controlled drug release and enhanced tissue regeneration. However, challenges, including long-term biocompatibility and large-scale synthesis, necessitate further research. Potential future directions encompass theranostics, immunomodulation, neural interfaces, bioelectronic medicine, and expanding bioimaging capabilities. In summary, both CQDs and carbon 2D nanosheets hold promise to revolutionize biomedical sciences, offering innovative solutions and improved therapies in diverse biological contexts. Addressing current challenges will unlock their full potential and can shape the future of medicine and biotechnology.

## 1. Introduction

Carbon nanomaterials have garnered significant attention in the field of biomedicine due to their remarkable physicochemical properties and tenability [1,2,3]. Nanoscale carbon-based structures, which include carbon nanotubes (CNTs), graphene, and fullerenes, possess distinct characteristics such as high surface area mechanical strength, exceptional electrical and thermal conductivity, and versatile surface chemistry [4,5]. These properties make them highly appealing for a wide range of biomedical applications. The application of carbon nanomaterials in biomedicine holds great promise, especially in targeted drug delivery [6]. Medical imaging [7,8,9], biosensing [10,11,12], tissue engineering [13,14], cancer therapy, gene delivery [15,16], wound healing [17], and antimicrobial interventions [18]. One notable advantage of carbon nanomaterials is their ability to be customized and tailored to specific biological purposes. This allows for improved biocompatibility. Targeted interactions with biological entities. In addition, controlled the release of therapeutic agents. In drug delivery, carbon nanomaterials can be designed to encapsulate diverse drugs, peptides, or nucleic acids. This enables precise and localized delivery to specific cells or tissues [19,20,21,22]. By adopting this targeted approach, adverse effects on non-targeted areas can be minimized while increasing treatment efficiency to enhance patient outcomes. Additionally. The unique optical and magnetic properties of carbon nanomaterials prove beneficial as contrast agents in various medical imaging techniques. This offers improved disease detection and monitoring by enhancing resolution and sensitivity [23,24,25,26,27,28].

Carbon nanomaterials have also shown great potential in biosensing applications [29,30]. Through surface functionalization, these nanomaterials can be designed to interact specifically with biomolecules, pathogens, or disease markers, leading to highly sensitive and selective detection platforms. Additionally, their incorporation into tissue engineering scaffolds allows for improved cell adhesion, proliferation, and tissue regeneration, presenting promising avenues for tissue repair and regeneration.

In the realm of cancer therapy, carbon nanomaterials have emerged as innovative candidates for various approaches [31]. Photothermal therapy (PTT) exploits their ability to convert absorbed light into heat, leading to the selective destruction of cancer cells upon laser irradiation [32]. On the other hand, photodynamic therapy (PDT) leverages their capacity to generate reactive oxygen species when exposed to light, inducing cell death in cancerous tissues [33].

Furthermore, carbon nanomaterials can play a crucial role in gene therapy [34], acting as carriers for delivering genetic material to target cells [35]. This opens up possibilities for treating genetic disorders and other genetic-based diseases more effectively [36].

Despite their immense potential, the use of carbon nanomaterials in biomedicine also raises concerns regarding their biocompatibility, potential toxicity, and long-term effects [37,38]. Proper evaluation of safety and thorough regulatory measures are imperative to ensure their safe and effective integration into medical applications.

This review aims to explore the numerous medical applications of carbon nanomaterials and provide insights into the challenges and opportunities they offer in the pursuit of advanced the-rapeutic strategies [11,39,40]. The advancement of carbon nanomaterials in biomedicine has the potential to revolutionize medical research and healthcare, ushering in a new era of precision medicine and personalized treatment approaches. Through their use, a shift towards targeted treatments tailored to individual patients can be expected.

## 2. Carbon Quantum Dots in Biological Applications

Carbon quantum dots (CQDs) have emerged as a fascinating class of nanomaterials with unique properties, including small size, tunable photoluminescence, excellent biocompatibility, and surface functionalization capabilities [41,42,43,44]. These characteristics make CQDs attractive candidates for a wide range of biological applications. In this discussion, we will focus on the current results and advancements in utilizing carbon quantum dots in various biological contexts.

### 2.1. Imaging and Bioimaging

**Figure 1:**
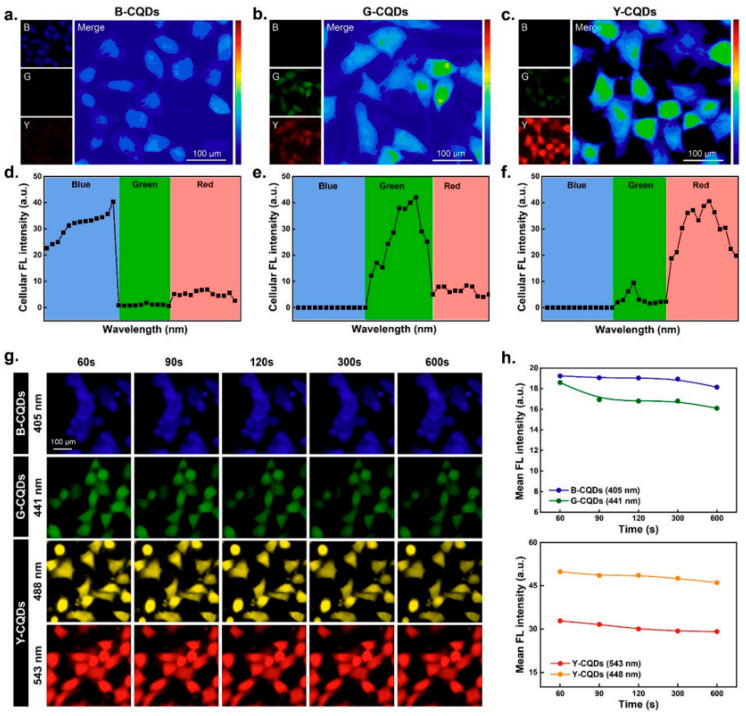
Multicolor bioimaging properties of CQDs. Confocal laser scanning microscopy (CLSM) and pseudo-color merged images of HepG2 cells incubated with (**a**) B-CQDs, (**b**) G-CQDs, and (**c**) Y-CQDs for 4 h. Images were acquired using 405 nm, 441 nm, and 543 nm excitation lasers for the respective CQDs. The color map shows the maximum (red) and minimum (blue) pixel densities in the merged image. (**d**–**f**) Represent the cellular fluorescence (FL) intensity of B-CQDs, G-CQDs, and Y-CQDs at different excitation wavelengths. (**g**) shows the photostability of B-CQDs, G-CQDs, and Y-CQDs under various excitation lasers over 10 min with (**h**) corresponding FL intensities. Scale bar: 100 μm. Reprinted with permission from Ref. [49].

Carbon quantum dots have shown exceptional potential as contrast agents in bioimaging applications [45]. Their tunable photoluminescence enables them to emit fluorescence in different regions of the electromagnetic spectrum, making them suitable for multiplexed imaging and real-time tracking of biological processes [46,47,48]. CQDs have been used in fluorescence microscopy, confocal imaging, and in vivo imaging, providing valuable insight into cellular dynamics and interactions [22,49]. Recent research has brought attention to the utilization of CQDs in precise imaging of particular biomolecules, such as proteins or nucleic acids, through the modification of the dot’s surface with suitable targeting components [22,50,51,52]. This approach allows for high specificity and sensitivity in detecting biomarkers associated with diseases, aiding in the early diagnosis and monitoring of pathological conditions (Figure 1).

### 2.2. Drug Delivery

Carbon quantum dots have shown promise as drug delivery vehicles due to their high surface area and ability to encapsulate therapeutic agents [53]. By functionalizing the surface with appropriate ligands, CQDs can effectively target specific cells or tissues, reducing off-target effects and improving drug delivery efficiency [54]. Current research is focused on exploring CQDs as carriers for chemotherapeutic drugs, gene therapy agents, and other therapeutic payloads, with encouraging results in enhancing treatment outcomes and reducing systemic toxicity [55].

### 2.3. Bio-Sensing and Diagnostics

The exceptional optical properties of carbon quantum dots make them ideal candidates for biosensing applications [56]. Researchers have developed CQD-based biosensors for detecting various analytes, such as glucose, ions, and pathogens [11,57,58,59]. Functionalization of CQDs with biomolecules or specific receptors enables the selective and sensitive detection of target analytes, contributing to the development of rapid and accurate diagnostic tools [60].

Carbon quantum dots (CQDs) have emerged as highly promising nanomaterials for biosensing applications due to their unique optical properties, biocompatibility, and surface chemistry. They offer numerous advantages, including high sensitivity, stability, and low toxicity, making them ideal candidates for various biosensors targeting glucose, biomolecules, DNA, and cancer biomarkers.

#### 2.3.1. Glucose Biosensors

**Figure 2:**
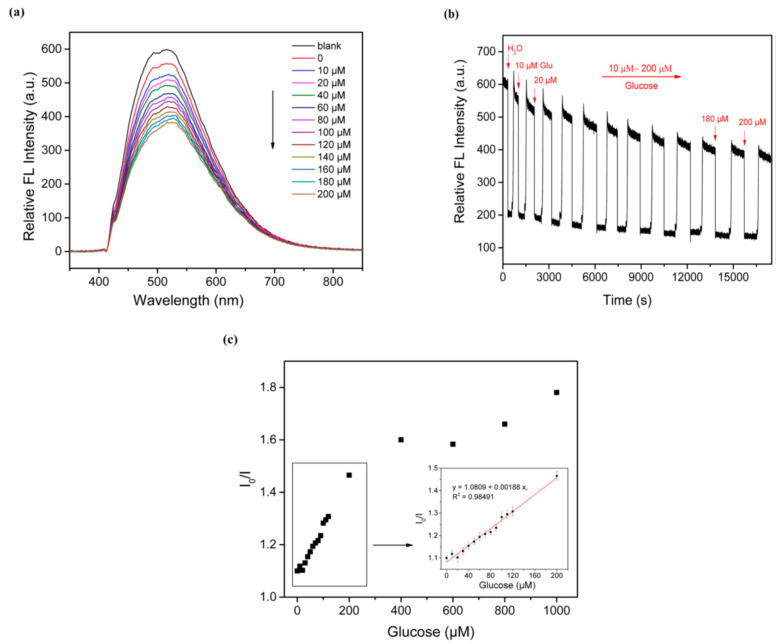
(**a**) This section presents the fluorescence spectra of the optical fiber sensor when exposed to glucose solutions of varying concentrations, ranging from 10 to 200 μM. (**b**) The diagram in this section illustrates the time sequence for detecting glucose using the optical fiber sensor. (**c**) The calibration curve provided here showcases the relationship between the concentration of glucose and the response of the optical fiber sensor, facilitating the determination of glucose levels. Reprinted with permission from Ref. [62], Elsevier, https://doi.org/10.1016/j.bios.2019.111760.

Glucose biosensors are essential for monitoring blood glucose levels in diabetic patients. CQDs have been utilized to design highly sensitive and selective glucose biosensors. For instance, Liu et al. (2018) developed a fluorescent glucose biosensor using N-doped CQDs with a high quantum yield of 45.4%. The sensor exhibited excellent glucose sensing performance with a detection limit of 5.98 μM [61,62] (Figure 2).

#### 2.3.2. Biomolecule Sensors

CQDs have shown promising results in detecting various biomolecules, such as proteins, enzymes, and metabolites. Eddin et.al. reported a novel CQD-based biosensor for the ultrasensitive detection of dopamine, an important neurotransmitter [63]. The sensor demonstrated a wide linear range and a low detection limit of 62 nM, indicating its potential for biomolecule sensing [64].

#### 2.3.3. DNA Sensors

CQDs have been integrated into DNA sensors for the detection of specific DNA sequences or genetic mutations associated with diseases. Shamsipur et al. developed a highly sensitive quantum dots-DNA nanobiosensor based on fluorescence resonance energy transfer for rapid detection of nanomolar amounts of human papillomavirus [65]. The sensor exhibited high sensitivity and selectivity, offering a reliable tool for early diagnosis [66].

#### 2.3.4. Cancer Biomarker Detection

CQDs have shown great promise in cancer biomarker detection, enabling early diagnosis and personalized treatments. Li et al. (2017) designed a CQD-based immunosensor for the ultrasensitive detection of carcinoembryonic antigen (CEA), a common cancer biomarker. The sensor demonstrated a low detection limit of 1.67 pg/mL and excellent specificity, showing potential for early-stage cancer diagnosis [67].

Overall, the current results demonstrate the tremendous potential of carbon quantum dots in biosensing applications. Their excellent performance in glucose biosensors, biomolecule sensors, DNA sensors, and cancer biomarker detection showcases their versatility and applicability in various healthcare settings. Continued research and optimization of CQD-based biosensors hold the promise of revolutionizing disease diagnosis, monitoring, and personalized medicine, contributing to improved patient outcomes and enhanced healthcare solutions.

### 2.4. Photodynamic Therapy (PDT)

Carbon quantum dots (CQDs) have emerged as a promising candidate for Photodynamic Therapy (PDT), an invasive treatment approach for cancer and microbial infections. PDT involves the use of photosensitizers that can produce reactive oxygen species (ROS) upon light activation, leading to cell death and the destruction of pathogens. CQDs exhibit unique properties that make them suitable for PDT applications, including their tunable photoluminescence, biocompatibility, and potential for targeted therapy.

#### 2.4.1. Photodynamic Therapy for Cancer

**Figure 3:**
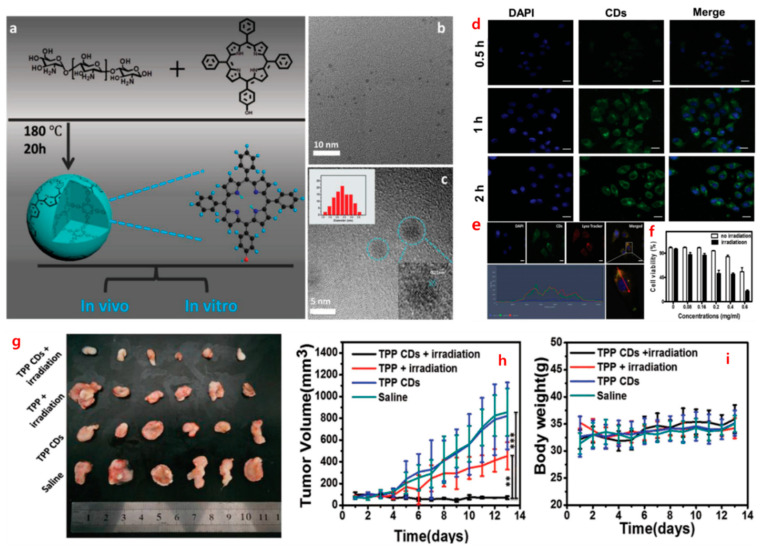
(**a**) This section outlines the synthetic pathway employed for the production of TPP CDs. (**b**) Transmission Electron Microscopy (TEM) images of TPP CDs are provided, offering insights into their morphology. (**c**) A high-resolution TEM image of TPP CDs is depicted, with an inset displaying the size distribution of the CDs. (**d**) Confocal Laser Scanning Microscopy (CLSM) images demonstrate the interaction between TPP CDs and HepG2 cells over different incubation periods. (**e**) A colocalization image exhibiting the co-localization of TPP CDs (green) and Lyso-Tracker (red) within the cells is presented. Scale bar: 20 μm. (**f**) Results from the MTT assay reveal the viability of cells following incubation with TPP CDs, with and without irradiation. (**g**) A photograph illustrating excised tumors on day 13 of the experiment is shown. (**h**) The tumor volume of mice over time is graphically represented, providing insights into tumor growth dynamics. (**i**) Changes in body weight of H22 cancer-bearing mice over time are plotted, serving as an indicator of overall health and treatment effects. Reprinted with permission from Ref. [68], 2016, WILEY-VCH Verlag GmbH & Co. KGaA, Weinheim. (Adv. Healthcare Mater. 2016, https://doi.org/10.1002/adhm.201600924).

CQDs have shown great potential in PDT for cancer treatment. Li et al. (2017) developed CQD-based nanocomposites with a porphyrin photosensitizer for enhanced PDT efficacy. The study demonstrated effective tumor suppression in a mouse model, highlighting the therapeutic potential of CQDs in cancer treatment [68]. Additionally, Li et al. (2020) utilized CQDs as a platform to co-deliver photosensitizers and chemotherapeutic drugs, achieving a synergistic effect for improved cancer treatment [69] (Figure 3).

#### 2.4.2. Photodynamic Therapy for Microbial Infections

CQDs have also shown promise in PDT for treating microbial infections. Hao et al. (2021) reported the use of CQDs as photosensitizers to treat drug-resistant bacterial infections. The study demonstrated effective antibacterial activity against various pathogens, including mixed *Staphylococcus aureus* and *Escherichia coli*-infected wounds in rats [70]. Moreover, CQDs have been combined with other antimicrobial agents, such as silver nanoparticles, to enhance the PDT effect and combat biofilm-related infections [71].

#### 2.4.3. Targeted PDT Using CQDs

One of the key advantages of CQDs is their potential for targeted therapy. Nicholas et al. (2015) developed folate receptor-targeted CQDs for specific cancer cell imaging and PDT. The study demonstrated enhanced cellular uptake and selective PDT-induced cancer cell death [72]. Similarly, Zhang et al. (2018) functionalized CQDs with hyaluronic acid for targeted PDT of CD44 receptor-overexpressed cancer cells, achieving improved treatment outcomes [73].

#### 2.4.4. Imaging and Monitoring during PDT

CQDs’ unique photoluminescence properties can be utilized for imaging and monitoring during PDT. Sun et al. (2019) designed CQD-based nanoprobes for real-time monitoring of PDT-generated ROS in cancer cells, enabling precise treatment control [74].

Carbon quantum dots hold significant promise as photosensitizers in Photodynamic Therapy for cancer and microbial infections. Their tunable properties, biocompatibility, and potential for targeted therapy make them highly attractive in the field of therapeutics. With continued research and optimization, CQD-based PDT could revolutionize cancer and infectious disease treatments, offering a non-invasive, targeted, and effective approach for improved patient outcomes.

### 2.5. Bioimaging-Guided Therapy

The unique combination of imaging and therapeutic capabilities of CQDs allows for bioimaging-guided therapy [75]. By using CQDs as imaging agents, researchers can precisely visualize the location and progression of diseases, guiding therapeutic interventions. Furthermore, CQDs can be engineered to carry therapeutic agents, enabling targeted drug delivery based on real-time imaging feedback [76].

Carbon quantum dots (CQDs) have shown immense promise as bioimaging-guided therapy agents, enabling targeted drug delivery based on real-time imaging and contributing to improved diagnostics and treatments for various diseases and medical conditions. Their unique optical properties, biocompatibility, and tunable surface chemistry make them ideal candidates for precise and personalized therapeutic approaches.

#### 2.5.1. Bioimaging Applications

**Figure 4:**
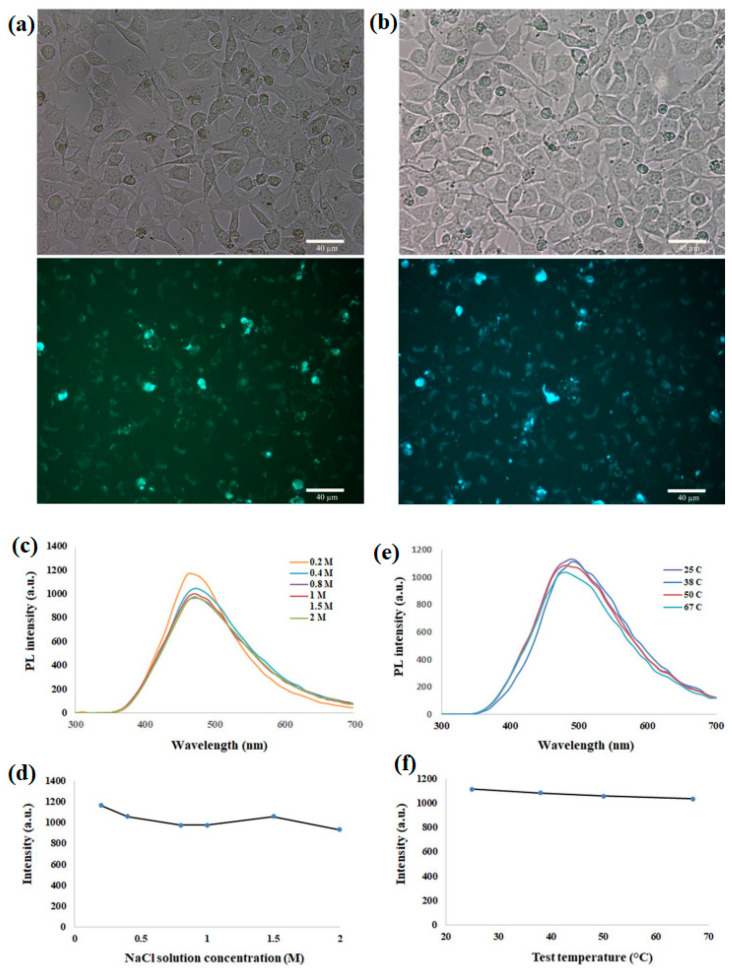
(**a**) Fluorescence microscopy images depict mouse C34/connective tissue-L929 cells labeled with CQDs as fluorescent probes. Bright-field images are positioned at the top, while corresponding fluorescent images are displayed at the bottom, representing cells treated with 1% CQDs. (**b**) Similar to (**a**), this section presents fluorescence microscopy images of Mouse C34/connective tissue-L929 cells treated with 3% CQDs, with bright-field images at the top and corresponding fluorescent images at the bottom. (**c**) Photoluminescence (PL) spectra of CQDs in ionic solutions with varying molarities are illustrated. (**d**) A diagram is provided, showcasing the maximum observed PL intensity of CQDs in different ionic solutions when excited at 300 nm. (**e**) PL spectra of CQDs at different temperatures are presented in this section. (**f**) A diagram representing the maximum observed intensity of CQDs at different temperatures when excited at 300 nm is depicted. Reprinted with permission from Ref. [78], 2022, Nature-Springer https://doi.org/10.1038/s41598-022-22518-0.

CQDs have been extensively used for bioimaging due to their excellent photoluminescence properties. Das et al. (2019) developed CQDs deposited capsules for fluorescence imaging-guided photothermal therapy of oral cancer, demonstrating enhanced tumor accumulation and effective therapy [77]. Additionally, CQDs have been used as contrast agents for bioimaging modalities such as magnetic resonance imaging (MRI) [78], computed tomography (CT) [79], and photoacoustic imaging (PAI) [80], offering comprehensive and real-time information for disease diagnosis and treatment planning (Figure 4).

#### 2.5.2. Targeted Drug Delivery

CQDs can be functionalized to target specific cells or tissues, enabling targeted drug delivery. Liu et al. (2020) developed a CQD-based drug delivery system for the targeted delivery of doxorubicin to breast cancer cells, leading to improved treatment efficacy and reduced side effects [81]. Furthermore, CQDs have been combined with other nanomaterials, such as liposomes and nanoparticles, to enhance drug delivery efficiency and therapeutic outcomes [82].

#### 2.5.3. Theranostic Applications

CQDs have emerged as theranostic agents, integrating diagnostic and therapeutic functionalities on a single platform. Qin et al. (2020) developed CQD-based nanocomposites for fluorescence imaging-guided photodynamic therapy, offering simultaneous diagnosis and treatment of tumors [83]. Additionally, CQDs have been utilized for imaging-guided gene therapy, combining visual feedback and gene delivery capabilities for precise treatment [84].

#### 2.5.4. Improved Diagnostics and Treatments

CQDs have contributed to improved diagnostics and treatments for various medical conditions. In neurodegenerative diseases, CQDs have been employed for early-stage diagnosis and monitoring of disease progression [85]. Moreover, CQDs have shown potential in infectious disease management, including bacterial and viral infections, by providing real-time information on disease location and response to treatment [86,87].

Carbon quantum dots have emerged as versatile and powerful tools in bioimaging-guided therapy, targeted drug delivery, and theranostic applications. Their unique properties enable improved diagnostics, precise treatments, and real-time monitoring of therapeutic outcomes. As research in this field continues, CQDs are expected to play a crucial role in transforming healthcare by offering personalized and effective solutions for a wide range of diseases and medical conditions.

### 2.6. Carbon Quantum Dots in Future Prospective Biological Applications Which Is Not Yet Done Research

Carbon quantum dots (CQDs) have shown tremendous promise in various biological applications, as discussed in previous research [88,89,90]. However, several potential future avenues remain unexplored and could significantly expand the scope of their use in biomedicine. This discussion focuses on the potential directions for future research on carbon quantum dots in biological applications that have not yet been extensively investigated.

#### 2.6.1. Theranostics

Future research could explore the integration of theranostic capabilities into carbon quantum dots [91]. Theranostics refers to the combination of diagnostic and therapeutic functions within a single nanomaterial. Developing CQDs that can simultaneously act as imaging agents for diagnostics while delivering therapeutic payloads could revolutionize personalized medicine. Such theranostic CQDs could enable real-time monitoring of treatment responses, allowing clinicians to adjust therapy regimens according to individual patient needs.

#### 2.6.2. Immunomodulation

**Figure 5:**
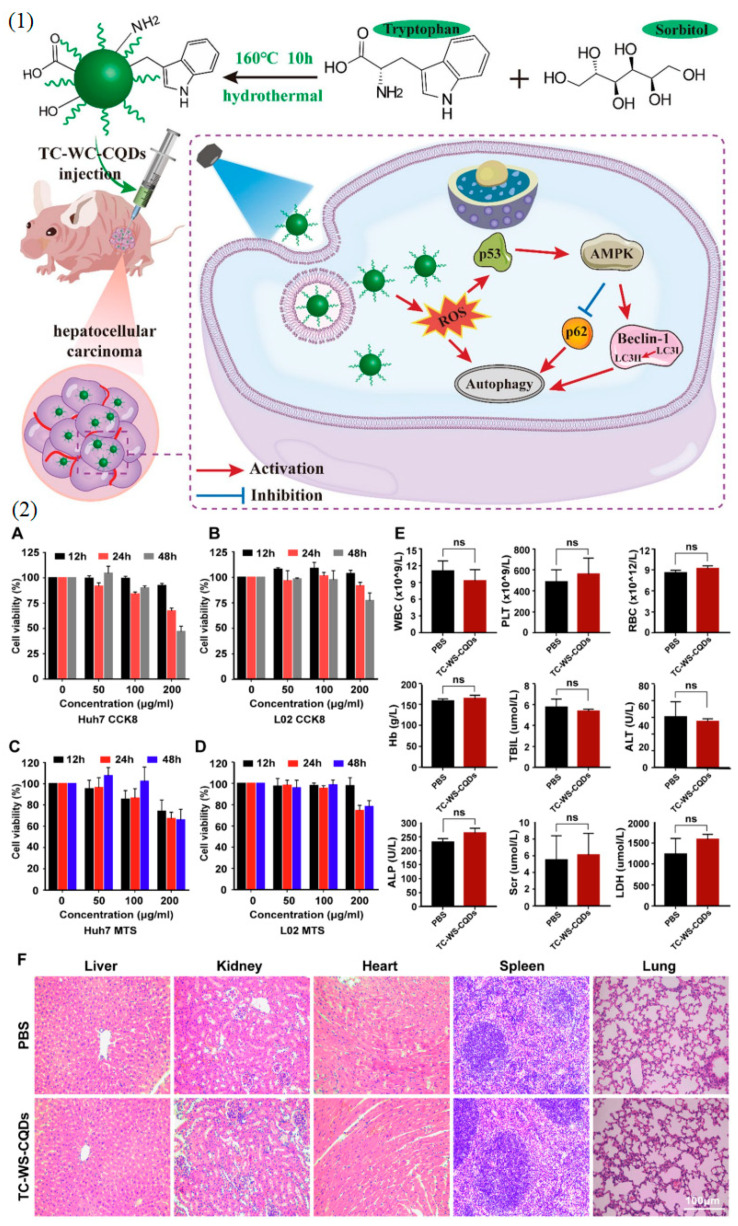
(**1**) Graphical representations delineating the synthesis procedure of Tryptophan-sorbitol-based carbon quantum dots (TC-WS-CQDs) and their theranostic mechanism against hepatocellular carcinoma are provided. (**2**) The biocompatibility of TC-WS-CQDs is evaluated through various assays. (**A**–**D**): Cell viability assays conducted on Huh7 cells and L02 cells using the CCK8 assay (**A**,**B**) and the MTS assay (**C**,**D**). Cells were seeded into 96-well plates at a density of 8 × 10^3^ cells/well. (**E**,**F**): Toxicity assessment of TC-WS-CQDs in BALB/nu mice (*n* = 5). Mice received intravenous injections of TC-WS-CQDs or PBS (300 μL) every 2 days. Blood and organs were collected on day 14 for analysis. (**E**): Complete blood count test and serum biochemistry results, including white blood cells (WBC), platelets (PLT), red blood cells (RBC), hemoglobin (Hb), total bilirubin (TBIL), alanine aminotransferase (ALT), alkaline phosphatase (ALP), serum creatinine (Scr), and lactate dehydrogenase (LDH). Data represent mean ± SD (*n* = 3 in (**A**–**D)**, *n* = 5 in (**E**)). Statistical significance analysis was performed using the Mann–Whitney test or unpaired *t* test where appropriate, with “ns” indicating not significant. (**F**): Histological evaluation of major organs from the mice. Reprinted with permission from Ref. [91] 2022, Springer-Nature, https://doi.org/10.1186/s12951-022-01275-2.

Investigating the immunomodulatory effects of carbon quantum dots is another unexplored area. CQDs’ unique surface properties and interactions with immune cells [92] could potentially be harnessed to modulate the immune system’s responses. Understanding how CQDs interact with immune cells and designing CQDs to enhance immune responses or suppress undesirable immune reactions could open up new avenues for immunotherapy and autoimmune disease treatments (Figure 5).

#### 2.6.3. Neural Interfaces and Neuroprotection

Carbon quantum dots’ biocompatibility and optical properties make them intriguing candidates for neural interfaces and neuroprotection applications [93]. Future research could explore the use of CQDs as neural probes or optogenetic tools to study and manipulate neural circuits. Additionally, investigating CQDs’ potential for enhancing neuroprotection and promoting neural tissue regeneration after injury or neurodegenerative diseases could have significant implications for neuroscience and neural engineering.

#### 2.6.4. Bioelectronic Medicine

Bioelectronic medicine is an emerging field that involves using electronic devices to modulate neural circuits and treat diseases [94]. Integrating CQDs into bioelectronic interfaces could provide unique advantages, such as enhanced biocompatibility, electrical conductivity, and optical sensing capabilities. Research in this area could lead to the development of novel bioelectronic devices for precise neuromodulation and targeted therapeutic interventions.

#### 2.6.5. Bioimaging beyond Visible Light

While CQDs have been extensively explored as fluorescent imaging agents in the visible light spectrum, their potential in other imaging modalities remains largely unexplored [95]. Research could focus on engineering CQDs to emit fluorescence in the near-infrared or even in non-optical modalities such as ultrasound or magnetic resonance imaging (MRI). These advancements could enable deeper tissue penetration, reduced auto-fluorescence, and improved image resolution for in vivo imaging.

#### 2.6.6. Drug-Resistant Pathogen Treatment

Addressing drug-resistant pathogens is a critical challenge in healthcare [96]. Future research could explore the use of carbon quantum dots in combination with antimicrobial agents to overcome drug resistance. CQDs’ unique properties could potentially enhance the efficacy of existing antimicrobial treatments or offer new mechanisms to combat drug-resistant infections [97].

### 2.7. Limitations of Carbon Quantum Dots in Biological Applications

While carbon quantum dots (CQDs) offer numerous advantages for biological applications, they also face certain limitations that need to be addressed to fully exploit their potential [98]. Some of the key limitations of CQDs in biological applications include:

#### 2.7.1. Size and Stability

The small size of CQDs can pose challenges in terms of stability and potential aggregation in biological environments [99]. Maintaining the stability of CQDs in various physiological conditions, such as temperature, pH, and ionic strength, is crucial to ensuring their long-term effectiveness and safety.

#### 2.7.2. Biocompatibility and Toxicity

Although CQDs are generally considered biocompatible, their potential toxicity remains a concern [100]. The exact mechanisms of CQD toxicity are not yet fully understood, and further research is required to comprehensively assess their impact on various cell types and biological systems.

#### 2.7.3. Long-Term Effects

The long-term effects of CQDs in biological systems are not well studied [101]. As these nanomaterials are relatively new, rigorous investigations are necessary to evaluate their biocompatibility over extended periods and assess any potential accumulative effects.

#### 2.7.4. Biodistribution and Clearance

**Figure 6:**
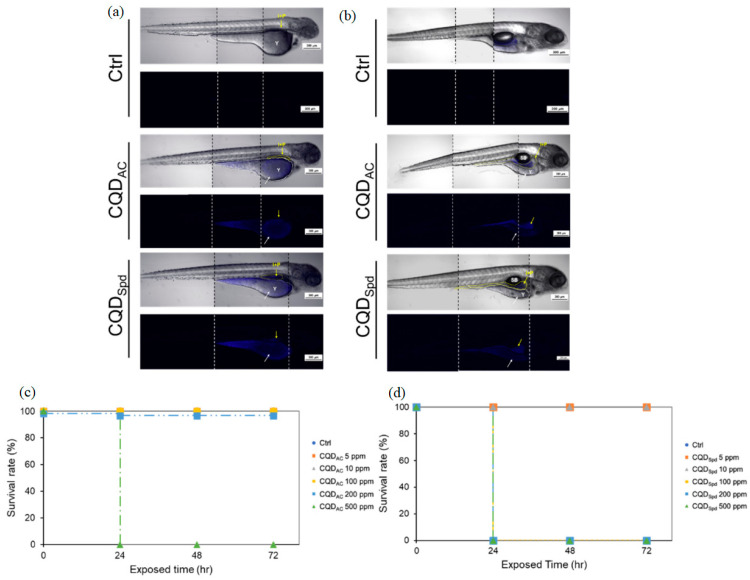
This section explores the distribution and bioaccumulation of CQDAC and CQDSpd through the observation of zebrafish eleutheroembryos. (**a**) Bright-field and fluorescence images depict the zebrafish eleutheroembryos after 24 h of exposure to CQDs. (**b**) Similarly, images captured 72 h after returning to normal conditions following exposure to CQDs are presented. The yellow arrow highlights the area containing the intestines (I) and the pancreas (P), while the white arrow indicates the location of the yolk sac (Y). Scale bar = 300 μm. Effective concentrations of CQDAC and CQDSpd in the fish eleutheroembryo acute toxicity (FEET) test. Survival rates of 96-h post-fertilization eleutheroembryos exposed to various concentrations of (**c**) CQDAC or (**d**) CQDSpd solutions over 72 h. Reprinted with permission from Ref. [100], MDPI; https://doi.org/10.3390/polym13101598.

Understanding the biodistribution and clearance pathways of CQDs is essential for their safe and effective use in biological applications [102]. Proper characterization of how CQDs are metabolized and eliminated from the body will help mitigate potential risks associated with their accumulation (Figure 6).

#### 2.7.5. Synthesis and Scalability

The large-scale synthesis of CQDs with consistent properties and quality remains challenging [103]. Improving the synthesis methods to ensure reproducibility, purity, and scalability is crucial for their widespread use in biomedicine.

#### 2.7.6. Specificity in Targeting

Achieving high specificity in targeting is critical for effective biomedical applications [104]. While functionalization allows for targeted interactions, the exact targeting mechanisms need further refinement to ensure precise and selective delivery to desired cells or tissues.

#### 2.7.7. Regulatory Challenges

As with any new nanomaterial, CQDs face regulatory challenges in terms of approval for medical applications [105]. Addressing safety concerns and obtaining regulatory approval requires comprehensive preclinical and clinical studies to demonstrate their efficacy and safety.

#### 2.7.8. Cost Effective

The cost of production and functionalization of CQDs can be a limiting factor in their widespread adoption in biomedical applications [106]. Reducing production costs while maintaining high-quality standards will be essential for their practical implementation.

#### 2.7.9. Photobleaching

CQDs, like many fluorescent probes, can undergo photobleaching, leading to reduced fluorescence intensity over time [107]. Developing more photostable CQDs or strategies to mitigate photobleaching effects is essential for long-term imaging applications.

Addressing these limitations through dedicated research, optimization of synthesis methods, and thorough preclinical studies [108] will pave the way for harnessing the full potential of carbon quantum dots in diverse biological applications. As the field progresses, the development of safe and effective CQD-based technologies will contribute significantly to the advancement of biomedical sciences and healthcare.

## 3. Carbon 2D Nanosheets in Biological Applications

Carbon 2D nanosheets, including graphene and graphdiyne, have garnered significant interest in biological applications due to their exceptional physicochemical properties. Here, we will discuss the current research and results focused on their potential roles in various biological contexts.

### 3.1. Graphene in Biosensing and Bioimaging

Graphene’s unique electrical and optical properties make it an excellent candidate for biosensing and bioimaging applications [109]. Graphene-based biosensors have demonstrated high sensitivity and specificity in detecting biomolecules, such as DNA, proteins, and various disease markers [110]. Additionally, graphene’s fluorescence quenching ability allows for sensitive detection in fluorescence-based assays [111]. In bioimaging, graphene’s photostability and biocompatibility make it suitable as a contrast agent for fluorescence imaging and photoacoustic imaging, offering enhanced resolution and sensitivity [112].

Researchers have developed graphene-based biosensors with enhanced selectivity through surface functionalization with specific receptors, antibodies, or aptamers [113]. These biosensors have been successfully applied in real-time monitoring of disease biomarkers and pathogen detection [114]. Graphene-based contrast agents have shown promising results in improving the resolution and accuracy of bioimaging techniques [115].

Carbon 2D nanosheets, including graphene, graphene oxide (GO), reduced graphene oxide (rGO), and graphedyne, have gained significant attention in biological applications, particularly in the detection of biomolecules such as DNA, proteins, and disease markers [116]. Their exceptional sensitivity and specificity, along with their unique physicochemical properties, have led to their successful integration into various fluorescence-based assays, fluorescence imaging, and photoacoustic imaging techniques [117].

#### 3.1.1. DNA Sensing

Carbon 2D nanosheets have shown great promise in DNA sensing applications. Kilic et al. (2016) developed a graphene-based electrochemical DNA sensor that demonstrated high sensitivity and specificity in the detection of specific DNA sequences related to cancer mutations [118,119]. Similarly, rGO has been utilized in fluorescence-based DNA sensors for the detection of genetic disorders, showcasing its capability for early diagnosis [120]. Graphdiyne has been utilized for DNA detection, enabling real-time monitoring of DNA-protein interactions [121].

#### 3.1.2. Protein Detection

**Figure 7:**
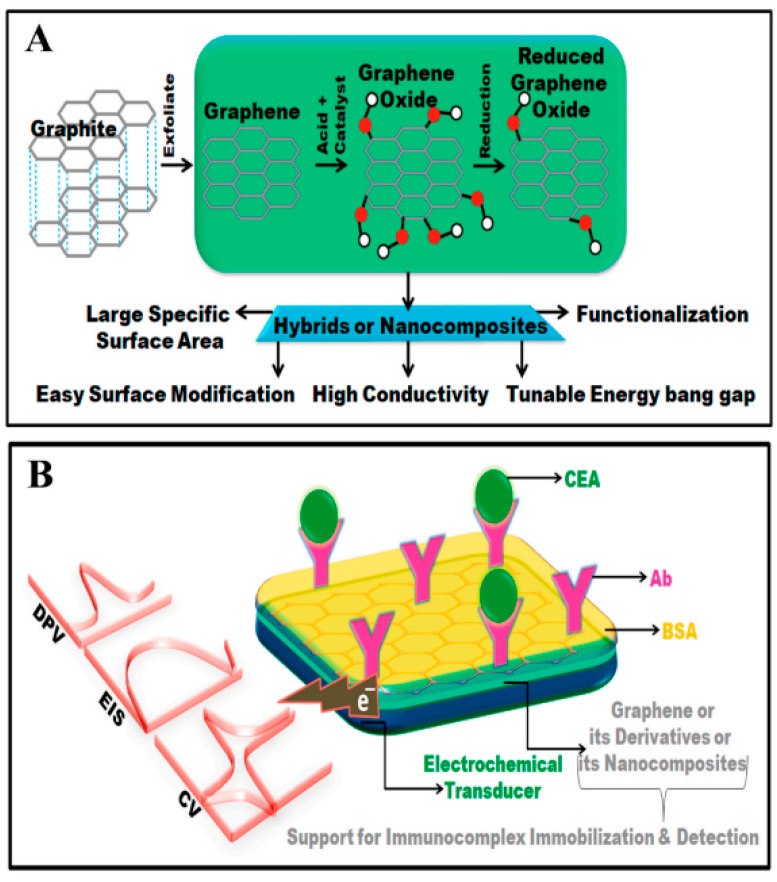
(**A**) This figure presents a schematic diagram outlining various methods for synthesizing graphene. (**B**) Graphical representations of devices constructed using graphene are depicted in this figure. Reprinted with permission from Ref. [124], 2022, Elsevier, https://doi.org/10.1016/j.biosx.2022.100189.

Graphene, graphene oxide, and graphdiyne have been employed for sensitive protein detection due to their large surface areas and excellent electron transfer properties [122]. Yu et al. (2021) designed a graphene-based aptamer biosensor for the specific detection of thrombin, an important blood-clotting protein, with high sensitivity [123] (Figure 7).

#### 3.1.3. Disease Marker Detection

Carbon 2D nanosheets have been utilized in the detection of various disease markers, facilitating early diagnosis and personalized medicine. Graphene has been incorporated into sensors for the detection of cancer biomarkers, such as carcinoembryonic antigen (CEA) [124]. Additionally, rGO has been used in electrochemical immunosensors for the detection of disease-related proteins, offering a rapid and sensitive diagnostic tool [125].

#### 3.1.4. Fluorescence Imaging and Photoacoustic Imaging

Graphene and GO have been explored as contrast agents in fluorescence imaging and photoacoustic imaging due to their strong fluorescence and photoacoustic signals [126]. Guo et al. (2018) developed a graphene oxide-based nanocomposite for fluorescence imaging of tumors, demonstrating high specificity in tumor targeting [127]. In photoacoustic imaging, graphene-based nanomaterials have been used as photoacoustic agents for cancer imaging, offering deep tissue penetration and high spatial resolution [128].

Carbon 2D nanosheets, including graphene, GO, rGO, and graphedyne, hold immense potential in biological applications, especially in biomolecule detection and imaging techniques. Their sensitivity and specificity in detecting DNA, proteins, and disease markers, along with their compatibility with fluorescence-based assays, fluorescence imaging, and photoacoustic imaging, make them valuable tools in advancing diagnostics and treatments for a wide range of diseases. Continued research and optimization of these nanosheets are expected to further enhance their performance and enable their widespread use in the biomedical field.

### 3.2. Graphdiyne in Tissue Engineering and Biomedical Devices

Graphdiyne, a 2D carbon allotrope, possesses a unique 2D porous structure with a high surface area and mechanical strength [129]. These properties make graphdiyne suitable for tissue engineering applications [130]. Graphedyne scaffolds have been designed to mimic the extracellular matrix, promoting cell adhesion, proliferation, and tissue regeneration. Additionally, the tunable porosity of Graphdiyne allows for controlled drug release, making it valuable for localized therapy [131].

Current Results: Initial studies have demonstrated the biocompatibility and potential of graphdiyne scaffolds for promoting tissue regeneration in bone [132], nerve [133], and cardiac tissues [134]. Graphdiyne’s ability to support cell growth and facilitate tissue repair offers promising opportunities in regenerative medicine [135].

#### 3.2.1. Graphdiyne Scaffolds for Tissue Engineering

Graphedyne scaffolds have garnered significant interest in tissue engineering due to their structural properties. Wang et al. (2020) reported the synthesis of porous graphdiyne scaffolds for bone tissue engineering. The scaffolds showed excellent mechanical properties and an interconnected porous structure, providing a suitable microenvironment for cell growth and tissue regeneration. Moreover, they demonstrated enhanced osteogenic differentiation of stem cells, indicating their potential for bone tissue engineering applications [136].

#### 3.2.2. Cell Adhesion and Proliferation

Graphdiyne’s unique surface properties promote cell adhesion and proliferation. Li et al. (2023) investigated the biocompatibility of graphdiyne and its potential for neural tissue engineering. The study demonstrated that graphdiyne supported the attachment and growth of neural stem cells, suggesting its potential for nerve tissue regeneration [135]. This property is crucial for developing tissue-engineered constructs that can integrate seamlessly with the host tissue.

#### 3.2.3. Controlled Drug Release

Graphdiyne’s controlled drug-release capabilities make it valuable for localized therapy and tissue repair. Jin et al. (2018) developed a graphdiyne-based drug delivery system for the controlled release of growth factors in tissue engineering. The study revealed that the graphdiyne carrier allowed sustained release of growth factors, promoting cell proliferation and tissue regeneration [137]. This controlled release behavior is essential for optimizing therapeutic efficacy and minimizing side effects.

#### 3.2.4. Biomedical Devices

Apart from tissue engineering, graphdiyne has also shown promise in biomedical device applications. Li et al. (2021) reported an ultrasensitive respiration sensor based on graphdiyne suitable for life and health monitoring. The sensor exhibited non-contact detection with high sensitivity, making it suitable for wearable biomedical devices for health monitoring and diagnostics [138]. Such devices hold great potential for personalized healthcare and remote patient monitoring.

Graphdiyne holds immense potential in tissue engineering and biomedical devices due to its unique properties, such as promoting cell adhesion, proliferation, and tissue regeneration. The controlled drug release capability of graphdiyne scaffolds makes them valuable for localized therapy and tissue repair. These features offer exciting opportunities in regenerative medicine, enabling the development of innovative strategies for tissue engineering, wound healing, and disease treatment. Continued research and optimization of graphdiyne-based materials and devices are expected to lead to breakthroughs in regenerative medicine, improving patient outcomes, and transforming healthcare.

## 4. Carbon 3D Nanosheets in Biological Applications

The biological applications of 3D carbon nanosheets, including 3D graphene foams, vertically aligned carbon nanosheets, carbon nanosheet aerogels, and 3D graphdiyne nanosheets, were still in their early stages. While these materials show great potential in various fields such as energy storage, catalysis, and gas separation, their applications in biology are yet to be extensively explored. Here is a scientific discussion outlining the potential and current results of these 3D carbon nanosheets in biological applications:

### 4.1. D Graphene Foams

3D graphene foams possess a high surface area, mechanical flexibility, and excellent electrical conductivity, making them attractive for biological applications. The unique porous structure of graphene foams provides a large surface area for functionalization with biomolecules, enabling their use in biosensing, drug delivery, and tissue engineering. Functionalized graphene foams have shown potential as biosensors for detecting specific biomarkers due to their high sensitivity and biocompatibility [139]. Additionally, their large surface area can facilitate the loading and delivery of therapeutic agents in drug delivery systems [140]. However, further research is needed to understand the biocompatibility and long-term effects of graphene foams in biological environments.

3D graphene foams have emerged as a versatile and promising material in various biomedical applications due to their unique properties, such as their large surface area, excellent mechanical strength, and high electrical conductivity. These foams offer numerous advantages, including efficient immobilization of biomolecules, enabling their use in biosensing, drug delivery, and tissue engineering applications. Their 3D porous structure provides ample space for loading and delivering therapeutic agents, making them ideal candidates for drug delivery systems. Additionally, 3D graphene foams have been explored as biosensors for detecting specific biomarkers with high sensitivity and selectivity.

#### 4.1.1. Biosensing Applications

3D graphene foams have shown great potential as biosensors for detecting specific biomarkers in various diseases. Liu et al. (2020) developed a 3D graphene foam-based electrochemical biosensor for the ultrasensitive detection of glucose, a critical biomarker for diabetes. The sensor exhibited a wide linear range and low detection limit, highlighting its efficacy for biosensing applications [141].

#### 4.1.2. Drug Delivery Systems

The large surface area and porous structure of 3D graphene foams allow efficient loading and delivery of therapeutic agents in drug delivery systems. Ezzati et al. (2020) reported the fabrication of a 3D graphene foam-based drug delivery system for the sustained release of anticancer drugs. The foam provided a high drug loading capacity and demonstrated controlled release profiles, enhancing the therapeutic efficacy of the drug [140].

#### 4.1.3. Tissue Engineering

**Figure 8:**
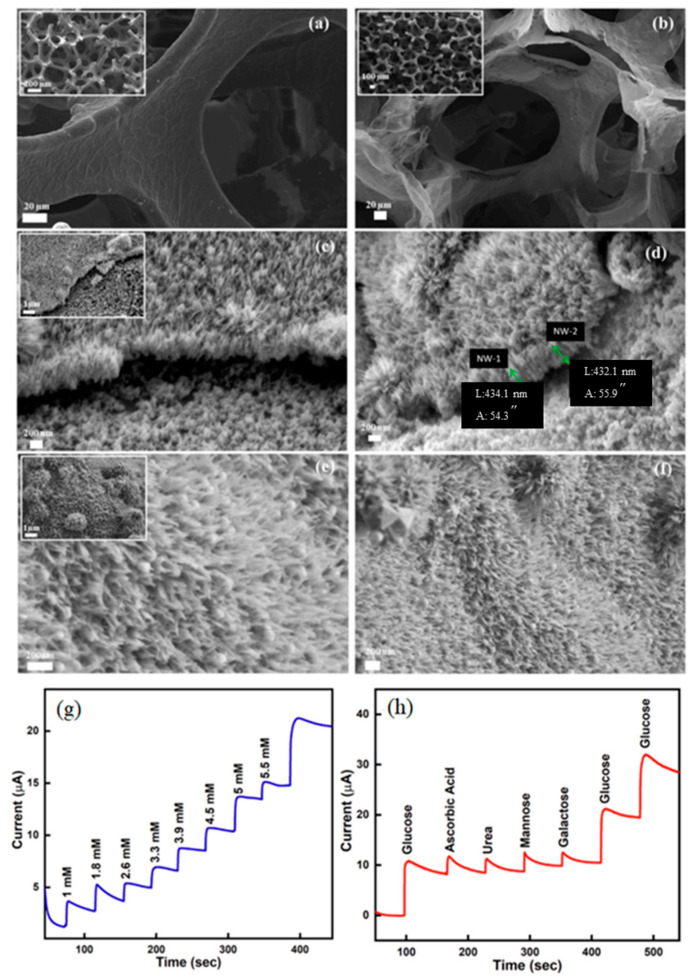
SEM images depict the morphologies of various materials: (**a**,**b**) Graphene foam (GF) is shown before and after nickel etching. (**c**,**d**) Hierarchical nanostructures (HNC) are observed on GF, with the upper portion depicting the growth of HNC and the lower portion showing GF covered with iron-oxide nanoparticles (NPs). (**e**,**f**) The nanocomposite electrode, in its final form as HNC/CS/GOx, is displayed after coating with chitosan (CS) and glucose oxidase (GOx). Insets provide lower-magnification images of each sample. The chronoamperometric response of the ITO/HNC/CS/GOx electrode towards successive additions of glucose and specific interfering molecules is presented. Chronoamperometric response of the ITO/HNC/CS/GOx electrode to successive glucose additions (**g**) and various interfering molecules (**h**). Reprinted with permission from Ref. [139], 2022, Elsevier, https://doi.org/10.1016/j.jallcom.2021.162688.

In tissue engineering, 3D graphene foams have been used as scaffolds to promote cell adhesion, proliferation, and tissue regeneration. Bahrami et al. (2019) developed a 3D graphene foam scaffold for cardiac tissue engineering. The scaffold exhibited excellent biocompatibility and enhanced cardiomyocyte adhesion and growth, demonstrating its potential for cardiac tissue repair [142] (Figure 8).

#### 4.1.4. Targeted Drug Delivery

Functionalization of 3D graphene foams allows targeted drug delivery to specific tissues or cells. Esmaeili et al. (2023) reported the development of a targeted drug delivery system based on folic acid-functionalized 3D graphene foam. The system showed enhanced cellular uptake and specific drug release in folate receptor-overexpressing cancer cells, offering a promising strategy for targeted cancer therapy [143].

3D graphene foams have demonstrated their immense potential in biosensing, drug delivery, and tissue engineering applications. Their unique 3D porous structure, along with the ability to immobilize biomolecules, enables sensitive and specific biosensing. Moreover, their large surface area and controllable drug release profiles make them excellent candidates for drug delivery systems. In tissue engineering, 3D graphene foams provide a favorable microenvironment for cell adhesion and tissue regeneration. Continued research and optimization of 3D graphene foams are expected to unlock further opportunities in the field of biomedicine, offering improved diagnostics and treatments for a wide range of diseases and medical conditions.

### 4.2. Vertically Aligned Carbon Nanosheets

Vertically aligned carbon nanosheets offer efficient electron and ion transport pathways, making them promising for biological applications requiring rapid charge transfer, such as bio electrochemical systems. For example, vertically aligned carbon nanosheets have been explored as electrode materials for biofuel cells and biosensors, demonstrating enhanced performance due to their unique nanostructure [144]. The vertical orientation of the nanosheets provides a direct pathway for electron transfer, enabling efficient electrochemical reactions in bio electrochemical devices.

Vertically aligned carbon nanosheets (VACNs) have garnered significant attention as promising bio electrochemical systems, particularly as electrode materials for biofuel cells and biosensors. Their unique properties, such as high surface area, excellent electrical conductivity, and favorable electrochemical characteristics, make them ideal candidates for various applications in bioelectrochemistry.

## 5. Biofuel Cells

VACNs have shown great potential as electrode materials for biofuel cells, which convert the chemical energy of biofuels into electrical energy through enzymatic reactions. Amade et al. (2016) reported the development of a high-performance biofuel cell using VACNs as the electrode material. The VACN-based biofuel cell demonstrated efficient electron transfer and high catalytic activity, leading to enhanced power generation [145].

### 5.1. Biosensors

VACNs have been utilized in biosensors for the sensitive and selective detection of various analytes. Termeh-Yousefi et al. (2015) designed a glucose biosensor based on VACNs, incorporating glucose oxidase as the biorecognition element. The biosensor exhibited excellent sensitivity and fast response times for glucose detection, showing its potential for medical and environmental applications [146].

### 5.2. Electrochemical Reactions in Bioelectrochemical Devices

In bioelectrochemical devices, VACNs play a crucial role in facilitating electrochemical reactions. Zhang et al. (2022) investigated VACNs as a hybrid bioanode for microbial fuel cells. The VACN-based bioanode exhibited high electrical conductivity and efficient biofilm formation, resulting in improved power generation with high performance [147].

### 5.3. Enzyme Immobilization

VACNs offer a suitable platform for enzyme immobilization, enhancing the stability and activity of enzymes in bioelectrochemical systems. Jiang et al. (2021) developed an enzyme-based biofuel cell using VACNs as the supporting material for enzyme immobilization. The VACNs provided a stable microenvironment for the enzymes, leading to enhanced catalytic activity and biofuel cell performance [148].

Vertically aligned carbon nanosheets have emerged as promising electrode materials in bioelectrochemical systems, facilitating efficient electrochemical reactions and offering opportunities in biofuel cells and biosensors. Their unique properties, including high electrical conductivity and a large surface area, make them ideal for enzyme immobilization and electron transfer processes. As research in this field continues, VACNs are expected to contribute significantly to the development of advanced bioelectrochemical devices, enabling sustainable energy generation and sensitive biosensing for various applications.

## 6. Carbon Nanosheet Aerogels

Carbon nanosheet aerogels exhibit exceptional mechanical strength and thermal stability, making them suitable candidates for biological applications that require lightweight and mechanically robust materials. While research on their specific applications in biology is limited, their properties make them potentially useful in tissue engineering scaffolds and lightweight structural materials in biomedical devices [149]. The interconnected 3D network of carbon nanosheets in aerogels provides a suitable environment for cell attachment and growth, making them potentially valuable in regenerative medicine.

Carbon nanosheet aerogels have emerged as a highly promising material with versatile applications in tissue engineering scaffolds and lightweight structural components for biomedical devices. Their unique properties, including high porosity, large surface area, and excellent mechanical strength, make them ideal candidates for various biomedical applications.

### 6.1. Tissue Engineering Scaffolds

Carbon nanosheet aerogels have been extensively studied as tissue engineering scaffolds due to their biocompatibility and ability to promote cell attachment and growth. Sala et al. (2018) developed a carbon nanosheet aerogel-based scaffold for nerve tissue engineering. The scaffold exhibited excellent biocompatibility, enhanced neuron adhesion, and proliferation of neurites, making it a promising platform for neurite regeneration [150].

### 6.2. Lightweight Structural Materials in Biomedical Devices

Carbon nanosheet aerogels have shown significant potential as lightweight structural materials for biomedical devices. Yang et al. (2021) fabricated a carbon nanosheet aerogel-based flexible strain sensor for wearable health monitoring applications. The sensor demonstrated excellent sensitivity and stability, highlighting the aerogel’s suitability for biomedical device integration [151].

### 6.3. Drug Delivery

Carbon nanosheet aerogels have also been explored for drug delivery applications. Lim et al. (2020) developed a model drug-coenzyme Q10-loaded carbon nanosheet aerogel. The aerogel exhibited a high drug loading capacity with a slow and sustained release profile, providing a promising approach for localized and controlled drug delivery [152].

### 6.4. Regenerative Medicine

Due to their unique properties and biocompatibility, carbon nanosheet aerogels hold promise in regenerative medicine. Atya et al. (2021) investigated carbon nanosheet aerogels as scaffolds for cardiac tissue engineering. The aerogels supported cardiomyocyte adhesion and growth, offering potential applications in cardiac regeneration [153].

**Figure 9:**
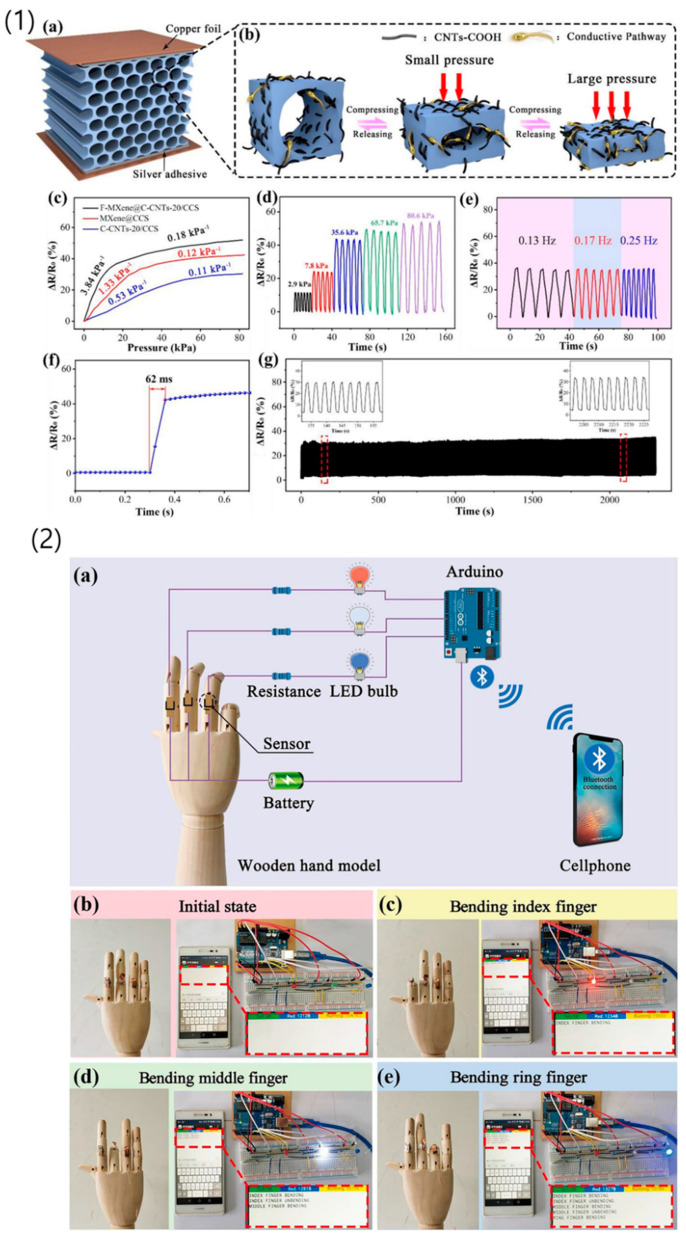
(**1**) This section comprises: (**a**) Schematic illustrations depicting the structure of the F-MXene@C-CNTs-20/CCS aerogel-based sensor and the working mechanism of the sensor. (**b**) Relative resistance changes of the F-MXene@C-CNTs-20/CCS, MXene@C-CNTs-0/CCS, and C-CNTs-20/CCS aerogel-based sensors with increasing applied pressure. (**c**) Response tests of the F-MXene@C-CNTs-20/CCS sensor under different stresses and frequencies. (**d**) Response time of the sensor. (**e**) Stability of the sensor during 1000 loading-unloading cycles at a compression strain of 30%. (**f**) Sensor response time. (**g**) Sensor stability over 1000 loading-unloading cycles at a 30% compression strain. Insets show the magnified response curves between 132–158 s and 2201–2226 s, respectively. (**2**) This section includes: (**a**) Schematic illustration of the Arduino microcontroller connected to a circuit for detecting finger movements and sending messages to a cellphone via Bluetooth connection. (**b**–**e**) Photographs of the wooden hand model in different states: initial state, bending index finger, bending middle finger, and bending ring finger. Corresponding photographs show messages from the Arduino microcontroller displayed on the cellphone and LED bulbs emitting red, white, and blue lights, respectively [151]. Reprinted with permission from Ref. [151], 2021, Elsevier, https://doi.org/10.1016/j.cej.2021.130462.

Carbon nanosheet aerogels have demonstrated great potential in tissue engineering scaffolds, lightweight structural materials for biomedical devices, and drug delivery systems. Their unique properties enable cell attachment, growth, and efficient drug loading and release, making them valuable in regenerative medicine and advanced biomedical applications. Continued research and optimization of carbon nanosheet aerogels are expected to unlock further possibilities in biomedical engineering, offering innovative solutions for tissue repair, drug delivery, and biomedical device development (Figure 9).

## 7. 3D Graphdiyne Nanosheets

3D graphdiyne nanosheets with tunable porosity and enhanced surface area could find applications in biological gas storage and separation. For instance, they might be utilized for the controlled release of gases in biomedical applications or as gas separation membranes [154]. However, their specific biological applications are yet to be extensively explored.

3D graphdiyne nanosheets have emerged as a promising material with exciting potential in biomedical applications, particularly in controlled gas release and gas separation membranes. Their unique structure and properties make them ideal candidates for various biomedical and environmental challenges.

### 7.1. Controlled Release of Gases in Biomedical Applications

Graphdiyne nanosheets have shown promise in controlled gas release applications for biomedical purposes. Min et al. (2020) developed a 3D graphdiyne-based nanosystem for controlled oxygen release. The nanosystem exhibited efficient oxygen loading and controllable release, making it a potential candidate for oxygen therapy in hypoxic conditions [155].

### 7.2. Gas Separation Membranes

Graphdiyne nanosheets have also been explored for gas separation applications in biomedical settings. Rezaee et al. (2020) fabricated a 3D graphdiyne-based membrane for CO_2_ separation. The membrane demonstrated excellent selectivity and permeability for CO_2_ separation, offering a promising solution for carbon capture and sequestration in the healthcare and environmental sectors [156].

### 7.3. Biomedical Gas Sensors

Graphdiyne nanosheets have shown potential in biomedical gas sensing applications. Li et al. (2021) developed a 3D graphdiyne nanowire for ultrasensitive detection of toxic gases. The nanowire sensor exhibited high sensitivity and selectivity, suggesting its applicability in real-time gas monitoring for medical diagnostics and safety [157].

### 7.4. Drug Delivery and Controlled Gas Release

3D graphdiyne nanosheets have also been studied for drug delivery applications, utilizing their gas storage capabilities. Tabandeh et al. (2021) reported a 3D graphdiyne-based nanosheet system for dual gas storage and drug delivery. The nanosheets demonstrated efficient gas storage and controlled drug release, offering potential therapeutic applications [158].

3D graphdiyne nanosheets show great promise in biomedical applications, particularly in controlled gas release and gas separation membranes. Their unique structure and properties enable efficient gas storage, controlled release, and selective gas separation, making them valuable tools in oxygen therapy, carbon capture, gas sensing, and drug delivery. Continued research and optimization of 3D graphdiyne nanosheets are expected to unlock further possibilities in biomedical engineering, providing innovative solutions to address medical and environmental challenges.

## 8. Carbon Nanosheet Composites

While carbon nanosheet composites have shown promise in enhancing mechanical, thermal, and electrical properties for various applications, their use in biology is not as well established. Composite materials incorporating carbon nanosheets could potentially be designed to improve the mechanical properties of tissue engineering scaffolds or enhance the electrical conductivity of bioelectrochemical devices [159]. However, more research is needed to determine their biocompatibility and suitability for specific biological applications.

As the field of nanomaterials advances, researchers are likely to explore the potential of 3D carbon nanosheets in various biological applications. However, it is essential to note that biological applications of nanomaterials require rigorous investigation of their biocompatibility, toxicity, and interactions with living systems to ensure their safe and effective use.

### 8.1. Tissue Engineering Scaffolds

**Figure 10:**
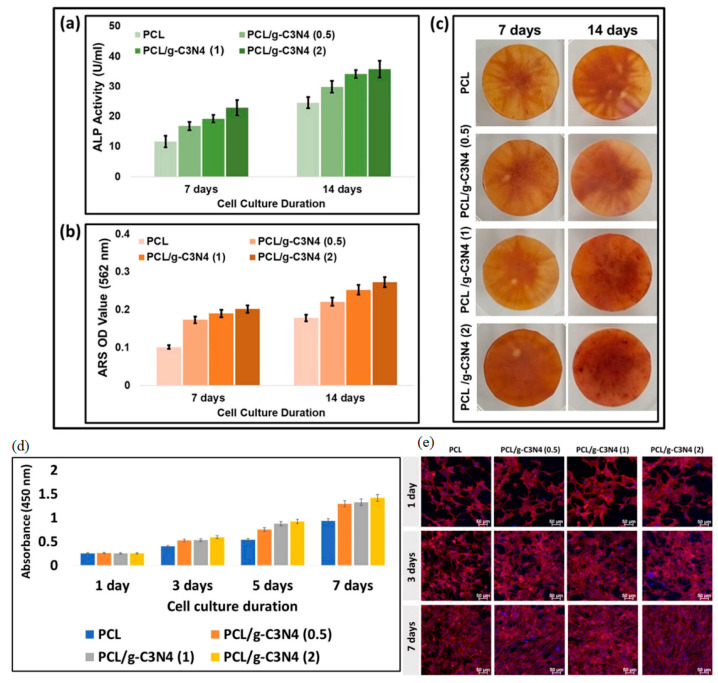
Cell viability assays and morphology staining were conducted on MC3T3-E1 cells cultured on PCL and composite PCL/g-C3N4 (0.5, 1, and 2 wt%) nanofiber scaffolds. (**a**) Cell viability was assessed using the CCK-8 assay, with the data presented as the mean ± standard deviation (*n* = 3). (**b**) Confocal Laser Scanning Microscopy (CLSM) images were captured at 1, 3, and 7 days. The nuclei and cytoplasm were stained with DAPI (blue) and Rhodamine-Phalloidin (red), respectively, with a scale bar of 50 µm. (**c**) Digital images of ARS staining after 7 and 14 days of culture. Cell viability and morphology assay of MC3T3-E1 cells cultured on PCL and composite PCL/g-C3N4 (0.5, 1, and 2 wt%) nanofiber scaffolds. (**d**) CCK-8 assay results, and (**e**) CLSM images at 1, 3, and 7 days. The CCK-8 test data are presented as mean ± standard deviation (*n* = 3). In the CLSM images, nuclei are stained with DAPI (blue), and the cytoplasm is stained with Rhodamine-Phalloidin (red); scale bar = 50 µm. Reprinted with permission from Ref. [160], 2021, Elsevier, https://doi.org/10.1016/j.colsurfa.2021.126584.

Carbon nanosheet composites have been explored as tissue engineering scaffolds to promote cell attachment, proliferation, and tissue regeneration. Awasthi et al. (2021) developed a 3D-printed scaffold using carbon nanosheets and polycaprolactone (PCL) for bone tissue engineering. The composite scaffold demonstrated excellent biocompatibility, mechanical properties, and enhanced osteogenic differentiation of stem cells, making it a promising candidate for bone regeneration applications [160] (Figure 10).

In another study, Jing et al. (2017) fabricated a carbon nanosheet composite hydrogel for cardiac tissue engineering. The hydrogel showed good biocompatibility, electrical conductivity, and improved cardiac cell proliferation and differentiation, indicating its potential in cardiac tissue regeneration [161].

### 8.2. Enhanced Electrical Conductivity in Bioelectrochemical Devices

Carbon nanosheet composites have also been used to enhance the electrical conductivity of bioelectrochemical devices, such as biofuel cells and biosensors. Mahalingam et al. (2020) reported a doped graphene-nanosheet-based biofuel cell. The composite electrode exhibited improved electron transfer kinetics and enhanced power generation, suggesting its potential application in energy harvesting devices [162].

In the field of biosensors, Muthusankar et al. (2019) developed a carbon nanosheet composite-based electrochemical biosensor for glucose detection. The biosensor demonstrated high sensitivity, selectivity, and stability, offering a promising platform for glucose monitoring in diabetes management [163].

So, carbon nanosheet composites have shown great promise in tissue engineering and bioelectrochemical devices. Their unique properties, including excellent biocompatibility, mechanical strength, electrical conductivity, and surface area, make them attractive candidates for tissue scaffolds and improve the performance of bioelectrochemical devices. Continued research and development of carbon nanosheet composites are expected to advance their applications in regenerative medicine, biosensing, and energy harvesting, leading to innovative solutions for medical and environmental challenges.

## 9. Carbon Nanotubes and their Derivatives in Biological Applications

Carbon nanotubes (CNTs) and their derivatives have garnered significant interest in biological applications due to their unique properties, including high aspect ratios, large surface areas, and tunable surface chemistry. These properties make CNTs suitable for a wide range of biomedical applications, such as drug delivery, imaging, biosensing, and tissue engineering. However, it is essential to address their potential biocompatibility and toxicity concerns to ensure their safe use in biological systems. Here is a scientific discussion with current results on the applications of CNTs and their derivatives in biology, along with relevant references.

### 9.1. Drug Delivery

Carbon nanotubes can be functionalized to encapsulate and deliver therapeutic agents to specific target sites in the body, enabling controlled and targeted drug delivery. Various studies have demonstrated the potential of CNTs for delivering anticancer drugs, antibiotics, and other therapeutic molecules. The surface functionalization of CNTs allows for easy attachment of drug molecules and targeting ligands, enhancing their efficacy and reducing off-target effects [35].

### 9.2. Bioimaging

Due to their unique optical properties, CNTs can serve as contrast agents in bioimaging techniques. Functionalized CNTs can be loaded with imaging agents such as fluorescent dyes, quantum dots, or radioisotopes to enhance imaging resolution and sensitivity. Their strong near-infrared (NIR) absorption and emission properties make them particularly suitable for NIR imaging and photoacoustic imaging [164].

### 9.3. Biosensing

CNTs have been extensively explored as biosensors for the detection of various biomolecules, pathogens, and disease markers. Their high surface area and excellent electrical conductivity allow for sensitive and label-free detection of analytes. Functionalized CNTs can be engineered to interact specifically with target biomolecules, enabling rapid and accurate sensing platforms [165].

### 9.4. Tissue Engineering

CNTs and their derivatives can be incorporated into scaffolds for tissue engineering applications. The small size and physical characteristics similar to the natural environment around cells allow for cell attachment, growth, and the restoration of tissues. Furthermore, carbon nanotubes (CNTs) have the ability to encourage the transformation of stem cells into specialized types, making them appealing for the field of regenerative medicine [166].

### 9.5. Biocompatibility and Toxicity

While CNTs hold great promise in biological applications, concerns regarding their biocompatibility and potential toxicity persist. Extensive investigation has been carried out to comprehend the interactions between carbon nanotubes (CNTs) and biological systems, as well as to address any negative impacts. Researchers have explored functionalization and surface modifications to improve biocompatibility and minimize toxicity [167].

Carbon nanotubes (CNTs) and their derivatives play a crucial role in the field of biology, constantly evolving and improving to maximize their potential while ensuring safety measures are addressed. As this field evolves, it is vital to carefully contemplate the moral, regulatory, and safety implications associated with the medical applications of CNTs, ensuring their responsible and advantageous utilization.

## 10. Clinical and Practical Aspects

Nanomedicine, the integration of nanotechnology in medicine, has revolutionized healthcare by providing innovative solutions for diagnosis, treatment, and disease monitoring. Carbon-based nanomaterials, such as graphene, carbon nanotubes (CNTs), fullerenes, and nanodiamonds, are at the forefront of this advancement. Their unique properties—including high surface area, biocompatibility, and functional versatility—make them ideal for diverse biomedical applications. Additionally, carbon nanomaterials such as carbon quantum dots (CQDs) and carbon 2D nanosheets (e.g., graphene, MXenes, and graphdiyne) show immense promise in diagnostics, therapy, imaging, and tissue engineering. These materials have been extensively studied for their potential to enhance the precision and effectiveness of medical treatments. However, despite their promising applications, several critical clinical and practical aspects, such as toxicity, biocompatibility, functionalization stability, and regulatory approval, must be thoroughly addressed to ensure their safe and effective use in widespread clinical settings. Continued research and interdisciplinary collaboration are essential to overcome these challenges and fully realize the potential of CBNs in transforming modern healthcare.

### 10.1. Clinical Aspects

#### 10.1.1. Targeting Strategies

Efficient targeting is crucial for enhancing therapeutic efficacy while minimizing off-target effects, particularly in the clinical application of carbon-based nanomaterials (CBNs). Functionalizing CBNs with targeting ligands such as antibodies, peptides, or small molecules can significantly increase their specificity towards diseased tissues or cells, thereby improving the precision of drug delivery and therapeutic interventions. This targeted approach is essential for personalized medicine, as it allows treatments to be tailored to the unique molecular profile of an individual’s disease. For instance, carbon nanotubes (CNTs) and graphene oxide (GO) can be functionalized with folic acid to target cancer cells overexpressing folate receptors, thereby enhancing the selective delivery of chemotherapeutic agents [168]. Similarly, conjugating nanodiamonds with targeting peptides can facilitate the delivery of therapeutic genes specifically to tumor cells [169]). Moreover, using multifunctional targeting strategies, such as combining imaging agents with therapeutic molecules on a single nanomaterial platform, can provide real-time monitoring of treatment efficacy and disease progression [170]. These strategies underscore the importance of versatile and precise targeting mechanisms in the clinical deployment of CBNs, highlighting the need for ongoing research to optimize their effectiveness and safety in personalized medical applications.

#### 10.1.2. Large-Scale Production

Practical implementation of carbon-based nanomaterials (CBNs) in clinical settings necessitates scalable and reproducible synthesis methods to ensure consistent quality and cost-effective mass production. Large-scale production is critical for the widespread adoption of CBNs in various biomedical applications, such as drug delivery, diagnostics, and imaging. Optimization of production processes involves refining techniques to achieve uniform size, shape, and functionalization of nanomaterials, which are essential for their reliability and effectiveness in clinical use. Methods such as chemical vapor deposition (CVD) for graphene and CNTs, high-pressure high-temperature (HPHT) synthesis for nanodiamonds, and bottom-up approaches for carbon quantum dots (CQDs) have shown promise in achieving scalable production [171,172].

Ensuring reproducibility across batches is crucial for regulatory approval and clinical application. Advanced techniques, such as roll-to-roll processing for graphene films and continuous flow reactors for CNTs, are being developed to enhance the scalability and uniformity of CBN production [173]. Moreover, improving purification and functionalization processes ensures that the produced nanomaterials maintain their desired properties and biocompatibility, essential for safe clinical application [174]. Continued research and innovation in large-scale production technologies will play a pivotal role in bringing CBN-based therapies and diagnostic tools from the laboratory to the clinic, ultimately improving patient care and outcomes.

#### 10.1.3. Clinical Imaging

Carbon nanomaterials’ unique photoluminescence properties present significant opportunities for advanced clinical imaging, yet integrating them into existing imaging systems and proving their clinical value demands extensive research and validation. These materials have critical applications in imaging and diagnostics due to their distinct optical and electronic properties. For example, nanodiamonds exhibit fluorescence and can serve as contrast agents in imaging techniques for instance magnetic resonance imaging (MRI) and fluorescence microscopy [175]. Additionally, graphene and its derivatives have been used to develop highly sensitive biosensors that can detect various biomolecules and pathogens, enhancing diagnostic capabilities [176]. Carbon quantum dots (CQDs) and 2D carbon nanosheets such as MXenes and graphdiyne have also demonstrated promise in bioimaging due to their excellent photoluminescent properties, enabling real-time imaging of biological processes and facilitating early diagnosis and disease monitoring [177].

#### 10.1.4. Drug Delivery Challenges

Carbon nanomaterials exhibit promise as drug delivery carriers, although addressing challenges such as drug loading efficiency, release kinetics, and stability during circulation is imperative. Understanding their interactions with biological barriers and clearance mechanisms is crucial for designing effective drug delivery systems. Carbon-based nanomaterials (CBNs) have shown remarkable potential in drug delivery systems owing to their large surface area and amenability to functionalization with therapeutic agents. For instance, graphene oxide (GO) can adsorb drugs and facilitate controlled release, thereby enhancing the efficacy of treatments such as chemotherapy [178]. Functionalizing graphene with polyethylene glycol (PEG) enhances its dispersion in biological environments, mitigating toxicity and enhancing biocompatibility [178]. In cancer therapy, carbon nanotubes (CNTs) can be functionalized with targeting ligands and loaded with drugs to deliver chemotherapeutics directly to tumor cells, thereby minimizing side effects and improving therapeutic outcomes [179]. Moreover, CNTs can be employed in photothermal therapy (PTT), where they generate heat upon exposure to near-infrared (NIR) light, selectively eradicating cancer cells [179].

#### 10.1.5. Personalized Medicine

The tunable properties of carbon nanomaterials offer opportunities for personalized medicine approaches. Tailoring nanomaterials for individual patient needs, such as drug delivery with specific payloads or targeting distinct biomarkers, could revolutionize disease treatments [180]. In conclusion, carbon nanomaterials show significant potential in clinical applications. Addressing safety concerns, optimizing targeting strategies, obtaining regulatory approvals, and scaling up production are critical for unlocking their full clinical benefits. Advancements in carbon nanomaterials research have the potential to transform healthcare, leading to improved diagnostics, personalized therapies, and innovative biomedical solutions.

#### 10.1.6. Tissue Engineering

The exceptional mechanical properties of carbon-based nanomaterials (CBNs) render them ideal candidates for tissue engineering applications. Graphene and carbon nanotubes (CNTs) can be integrated into scaffolds to bolster their mechanical strength and electrical conductivity, thereby facilitating cell adhesion, growth, and differentiation. These attributes are particularly advantageous in regenerating bone, nerve, and cardiac tissues [181]. For instance, graphene-based scaffolds have demonstrated the capacity to support the growth and differentiation of stem cells, making them well-suited for tissue repair applications. Similarly, CNT-based scaffolds exhibit potential for enhancing the regeneration of neural tissues, given their ability to conduct electrical signals critical for nerve cell function [181].

### 10.2. Practical Aspects

#### Biocompatibility and Toxicity

The biocompatibility of carbon-based nanomaterials (CBNs) is a critical consideration for their clinical application, as highlighted in various studies. The toxicity profiles of CBNs can significantly vary based on factors such as material type, size, and functionalization [182]. For instance, unmodified carbon nanotubes (CNTs) have been shown to induce oxidative stress and inflammation, while functionalized CNTs generally exhibit reduced toxicity [182]. Long-term biocompatibility studies are essential for understanding the potential health risks associated with CBN use in clinical settings. Comprehensive in vitro and in vivo assessments are necessary to determine safe dosage levels and identify possible side effects [183]. Thorough safety evaluations are imperative before transitioning CBNs to clinical use, as certain carbon quantum dots (CQDs) and graphene derivatives have exhibited toxicity at high concentrations or with specific functionalizations, potentially leading to organ accumulation [184]. Moreover, the variability in synthesis and functionalization of carbon nanomaterials can significantly influence their biocompatibility, underscoring the need for rigorous testing on different cell types and in vivo models to establish a comprehensive safety profile [185].

## 11. Challenges and Future Prospects

Carbon-based nanomaterials (CBNs) hold tremendous potential for revolutionizing healthcare and medical applications due to their unique properties, such as their high surface area, tunable physicochemical characteristics, and biocompatibility. However, their translation into clinical practice is accompanied by several challenges that need to be addressed. Simultaneously, ongoing research promises exciting future prospects for advancing the field.

### 11.1. Challenges

#### 11.1.1. Safety and Toxicity

The safety and toxicity profile of carbon-based nanomaterials (CBNs) is a critical consideration for their widespread use in healthcare and medical applications. While CBNs offer remarkable properties for various biomedical applications, including drug delivery, imaging, and tissue engineering, concerns regarding their potential adverse effects on human health have been raised [185]. Studies have shown that the toxicity of CBNs can vary depending on factors such as size, surface functionalization, and exposure concentration [186]. For instance, unmodified carbon nanotubes (CNTs) have been associated with oxidative stress and inflammation, highlighting the importance of surface modification to reduce their toxicity [185].

Comprehensive assessment of CBN toxicity involves in vitro and in vivo studies to evaluate their biocompatibility, biodistribution, and long-term effects. Understanding the mechanisms underlying CBN-induced toxicity is crucial for mitigating potential risks and ensuring their safe use in clinical settings [186]. Furthermore, regulatory bodies such as the FDA and EMA require rigorous safety evaluations before approving CBN-based medical products for human use [186]. Collaborative efforts between researchers, regulatory agencies, and industry stakeholders are essential for addressing safety concerns and advancing the responsible development of CBN-based healthcare technologies.

#### 11.1.2. Standardization and Reproducibility

Achieving standardized synthesis protocols and ensuring reproducibility across different batches of carbon-based nanomaterials (CBNs) remain significant challenges in their clinical translation. The unique properties of CBNs, such as their high surface area, electrical conductivity, and biocompatibility, are highly dependent on precise control over their synthesis and functionalization processes [187]. Variability in production processes can lead to inconsistencies in material properties, which in turn can affect their performance in biomedical applications. For instance, the synthesis of graphene can vary in terms of the precursor materials used, the methods of exfoliation, and the types of functionalization, all of which can impact its electrical properties, biocompatibility, and toxicity [188]. Similarly, the production of carbon nanotubes (CNTs) involves different techniques such as chemical vapor deposition (CVD), arc discharge, and laser ablation, each yielding products with varying degrees of purity, diameter, and chirality [187].

To address these challenges, robust quality control measures must be developed and implemented. This includes establishing standardized protocols for the synthesis and functionalization of CBNs, as well as developing reliable analytical methods to characterize their properties consistently. Techniques such as Raman spectroscopy, electron microscopy, and thermal gravimetric analysis can be employed to ensure the uniformity of CBN batches [188]. Moreover, collaboration among researchers, industry stakeholders, and regulatory bodies is crucial to developing guidelines and standards that ensure the reproducibility and reliability of CBN-based medical products. The establishment of such standards not only facilitates the clinical translation of CBNs but also enhances their credibility and acceptance within the scientific community and regulatory agencies. Ultimately, achieving standardization and reproducibility will be pivotal in unlocking the full potential of CBNs in healthcare applications, paving the way for their safe and effective use in diagnostics, therapy, and tissue engineering.

#### 11.1.3. Biocompatibility and Immunogenicity

Despite their promising biocompatibility, carbon-based nanomaterials (CBNs) may still elicit immune responses or cause adverse reactions within biological systems. Understanding these interactions is critical for minimizing immunogenicity and enhancing biocompatibility, ensuring the safe and effective use of CBNs in clinical applications (Mocan et al., 2017). The immune response to CBNs can vary based on factors such as size, shape, surface charge, and functionalization. For instance, unmodified carbon nanotubes (CNTs) have been reported to induce inflammatory responses, oxidative stress, and cytotoxicity, primarily due to their fibrous structure and potential to generate reactive oxygen species (ROS) [189]. Conversely, functionalizing CNTs with biocompatible polymers or biomolecules can significantly reduce their immunogenicity and enhance their compatibility with biological tissues. Graphene and its derivatives, such as graphene oxide (GO), also present challenges in terms of biocompatibility. While GO’s high surface area and functional groups facilitate drug loading and targeting, they can also interact with cellular membranes and proteins, potentially triggering immune responses [190]. The extent of these interactions often depends on the degree of oxidation and the nature of the functional groups attached to the graphene surface.

To mitigate these issues, thorough characterization of CBNs’ physicochemical properties is essential. Techniques such as surface charge measurements, particle size analysis, and evaluation of functional groups can provide insights into how these materials interact with biological systems [189]. Additionally, in vitro and in vivo studies are crucial for assessing the immunogenic potential of CBNs and identifying any adverse effects on immune cells and tissues. Moreover, developing strategies to modulate the immune response to CBNs is important. For instance, coating CBNs with polyethylene glycol (PEG) or other biocompatible molecules can create a “stealth” effect, reducing recognition by the immune system and prolonging circulation time in the bloodstream [190]. This approach not only enhances biocompatibility but also improves the efficacy of CBN-based drug delivery systems.

So, while CBNs hold great potential for biomedical applications, understanding and managing their interactions with the immune system are paramount for their safe and effective clinical use. Continued research and development of strategies to reduce immunogenicity will pave the way for the successful integration of CBNs in various therapeutic and diagnostic modalities.

### 11.2. Future Prospects

#### 11.2.1. Advanced Drug Delivery Systems

Carbon-based nanomaterials (CBNs) offer promising opportunities for the development of advanced drug delivery systems due to their unique properties such as high surface area, functional versatility, and biocompatibility. These properties enable CBNs to be tailored for specific therapeutic applications, enhancing targeting capabilities and enabling controlled release profiles [191]. Functionalization of CBNs with various molecules, including drugs, targeting ligands, and polymers, allows for precise delivery to specific tissues or cells, improving treatment outcomes while minimizing side effects.

Graphene oxide (GO), for example, has been widely studied for its potential in drug delivery. Its large surface area and abundant functional groups enable the loading of a substantial amount of therapeutic agents. Furthermore, GO can be functionalized with polyethylene glycol (PEG) to improve its solubility and biocompatibility, allowing for prolonged circulation in the bloodstream and reduced immunogenicity [192]. This functionalization also facilitates the attachment of targeting ligands, such as antibodies or peptides, which can direct the GO to specific cell types, such as cancer cells, thereby enhancing the specificity and efficacy of the drug delivery system.

Carbon nanotubes (CNTs) have also shown great promise in drug delivery applications. Their cylindrical structure and high aspect ratio allow for efficient drug loading and transport. CNTs can be functionalized to improve their dispersibility in biological fluids and reduce their potential toxicity. Moreover, the functionalization of CNTs with targeting ligands can direct them to specific tissues or tumor sites, enabling targeted therapy. For example, CNTs functionalized with folic acid have been used to target cancer cells that overexpress folate receptors, improving the delivery and effectiveness of chemotherapeutic agents [192].

Another exciting application of CBNs in drug delivery is their use in combination therapies. By loading multiple therapeutic agents onto a single CBN platform, it is possible to achieve synergistic effects, improving treatment outcomes. For instance, CNTs and graphene can be used to co-deliver chemotherapy drugs and genes, providing a multifaceted approach to cancer treatment. This combination can help overcome drug resistance and enhance the overall efficacy of the therapy [192].

The controlled release of drugs is another critical aspect of advanced drug delivery systems. CBNs can be engineered to release their payload in response to specific stimuli, such as pH changes, temperature variations, or enzymatic activity. This allows for precise control over the timing and location of drug release, maximizing therapeutic efficacy and minimizing side effects. For instance, CBNs functionalized with pH-sensitive polymers can release their drug load in the acidic environment of a tumor, providing targeted and controlled delivery [191].

So, the functional versatility and unique properties of CBNs make them ideal candidates for advanced drug delivery systems. Their ability to be tailored for specific therapeutic applications, combined with their potential for targeted delivery and controlled release, offers significant advantages over traditional drug delivery methods. Continued research and development in this field will likely lead to new and innovative approaches for the treatment of various diseases, improving patient outcomes and quality of life.

#### 11.2.2. Diagnostic and Imaging Technologies

Carbon-based nanomaterials (CBNs) possess unique optical and electronic properties that position them as promising candidates for next-generation diagnostic and imaging technologies. The development of advanced imaging techniques relies heavily on materials that can enhance the sensitivity and specificity of detection methods, and CBNs have shown significant potential in this regard. Carbon quantum dots (CQDs) and graphene-based contrast agents, in particular, are at the forefront of research in improving modalities such as magnetic resonance imaging (MRI), fluorescence imaging, and photoacoustic imaging [193]. Carbon quantum dots (CQDs) are fluorescent nanoparticles with excellent photostability, biocompatibility, and tunable emission wavelengths. These properties make them ideal for applications in fluorescence imaging, where they can be used to label and track biological molecules and cells with high precision. CQDs exhibit strong and stable fluorescence, which enhances the resolution and contrast of imaging, enabling the detailed visualization of cellular processes and molecular interactions [194]. Additionally, their small size and ability to be functionalized with various biomolecules allow for targeted imaging of specific tissues or pathological sites.

Graphene and its derivatives, such as graphene oxide (GO) and reduced graphene oxide (rGO), also hold significant promise as contrast agents for MRI and other imaging modalities. Graphene-based materials can be functionalized with magnetic nanoparticles, such as iron oxide, to create hybrid contrast agents that combine the high surface area and functional versatility of graphene with the magnetic properties of iron oxide [193]. These hybrid materials can enhance the contrast in MRI scans, improving the detection and characterization of tumors and other pathological conditions.

In photoacoustic imaging, graphene-based materials have shown potential due to their strong optical absorption and efficient photothermal conversion. When irradiated with laser light, graphene absorbs the light energy and generates ultrasonic waves through the photoacoustic effect. These waves can be detected to create high-resolution images of biological tissues, providing valuable information about the structure and function of organs and tumors [194]. The high sensitivity and resolution of graphene-based photoacoustic imaging make it a powerful tool for the early diagnosis and monitoring of diseases. The integration of CBNs into imaging technologies also extends to multimodal imaging, where different imaging techniques are combined to provide complementary information about a biological system. For example, combining MRI with fluorescence or photoacoustic imaging can offer both anatomical and molecular insights, enhancing the overall diagnostic capability. Graphene-based nanomaterials, with their versatile functionalization potential, are well-suited for such applications, enabling the design of multifunctional imaging probes that can target specific biomarkers and provide comprehensive diagnostic data [193].

Finally, the unique optical and electronic properties of CBNs make them highly promising for enhancing next-generation diagnostic and imaging technologies. The ability to functionalize these materials for specific applications, combined with their excellent biocompatibility and stability, positions them as valuable tools in the detection and monitoring of diseases. Continued research and development in this field are expected to lead to significant advancements in medical imaging, improving the accuracy and effectiveness of diagnostics, and ultimately contributing to better patient outcomes.

#### 11.2.3. Tissue Engineering and Regenerative Medicine

Carbon-based nanomaterials (CBNs) have shown significant potential in tissue engineering and regenerative medicine due to their ability to mimic the structural and mechanical properties of the extracellular matrix (ECM). This mimicry is crucial for promoting cell adhesion, proliferation, and differentiation, which are essential processes for tissue regeneration and repair. Among the various types of CBNs, graphene, carbon nanotubes (CNTs), and carbon quantum dots (CQDs) have been extensively studied for their applications in developing scaffolds for tissue engineering [195]).

Graphene and graphene oxide (GO) are particularly promising due to their high surface area, mechanical strength, and biocompatibility. When used in scaffolds, these materials can provide a conducive environment for stem cells and other cell types to adhere and grow. Studies have shown that graphene-based scaffolds can support the growth and differentiation of stem cells into various tissue types, including bone, nerve, and cardiac tissues [184]. For instance, graphene’s conductive properties are beneficial for neural tissue engineering, where electrical conductivity is critical for the development and function of nerve cells. Similarly, CNTs have been used to enhance the mechanical properties of scaffolds. Their high tensile strength and flexibility make them ideal for reinforcing polymer-based scaffolds, providing the necessary support for tissue formation. CNTs can also be functionalized with bioactive molecules to improve cell attachment and proliferation. In nerve tissue engineering, CNT-based scaffolds have been shown to facilitate the regeneration of neural tissues by providing a conductive pathway that supports the growth and alignment of neurons, which is crucial for nerve repair and regeneration [195].

CQDs, on the other hand, offer unique advantages due to their small size, photoluminescent properties, and ease of functionalization. They can be incorporated into scaffolds to provide both structural support and real-time imaging capabilities. This dual functionality allows researchers to monitor tissue growth and scaffold degradation over time, providing valuable insights into the tissue regeneration process [184].

Despite the promising applications of CBN-based scaffolds in tissue engineering, several challenges need to be addressed to fully realize their potential. One of the primary concerns is the long-term biocompatibility and potential toxicity of these materials. While many CBNs have been shown to be biocompatible in short-term studies, more research is needed to understand their long-term effects in vivo. Additionally, the standardization of synthesis and functionalization protocols is crucial to ensuring the reproducibility and reliability of CBN-based scaffolds in clinical applications [195].

So, carbon-based nanomaterials hold significant promise for revolutionizing healthcare and medical applications, particularly in advanced drug delivery systems, diagnostic and imaging technologies, tissue engineering, and regenerative medicine. The unique properties of CBNs, such as high surface area, biocompatibility, and functional versatility, make them ideal candidates for these applications. However, challenges such as safety concerns, standardization, and understanding long-term effects must be addressed. Continued research efforts aimed at overcoming these challenges are essential for unlocking the full potential of CBNs. As these materials advance through the stages of research and development, their transformative impact on healthcare delivery and patient outcomes is expected to grow, paving the way for innovative and effective medical treatments [184].

## 12. Conclusions

Reviewing the application of carbon-based nanomaterials (CBNs) in healthcare reveals a promising landscape of innovation and potential. These materials, including graphene, carbon nanotubes (CNTs), fullerenes, nanodiamonds, carbon quantum dots (CQDs), and 2D carbon nanosheets, offer a versatile platform for addressing various healthcare challenges, spanning from diagnostics to therapy and tissue engineering.

Firstly, CBNs exhibit remarkable properties that make them suitable for biomedical applications. Their high surface area, mechanical strength, electrical conductivity, and biocompatibility render them ideal candidates for drug delivery, biosensing, imaging, and tissue regeneration. For instance, graphene and CNTs can enhance the mechanical properties of scaffolds in tissue engineering, promoting cell adhesion, growth, and differentiation. Moreover, the photoluminescent properties of CQDs and 2D carbon nanosheets enable real-time imaging of biological processes, facilitating early disease diagnosis and monitoring.

In drug delivery, functionalized CBNs offer precise and efficient targeting of therapeutic agents to specific tissues or cells, minimizing off-target effects and enhancing therapeutic efficacy. By conjugating targeting ligands onto CBNs, such as antibodies or peptides, researchers can tailor drug delivery systems to individual patient needs, ushering in the era of personalized medicine.

However, alongside the tremendous potential, several challenges must be addressed to facilitate the clinical translation of CBN-based healthcare technologies. Safety and toxicity remain primary concerns, necessitating comprehensive evaluations to ensure the biocompatibility and long-term safety of these materials. Furthermore, achieving scalable and reproducible synthesis methods is crucial for mass production and widespread clinical implementation. Large-scale production processes must be optimized to maintain the quality and consistency of CBNs, meeting regulatory standards for clinical use.

Regulatory approval is another critical aspect that cannot be overlooked. Regulatory bodies such as the FDA and EMA require robust evidence on the safety, efficacy, and biocompatibility of CBN-based healthcare products before granting approval for clinical use. Ethical considerations surrounding the long-term impact of nanomaterials on human health and the environment also warrant careful examination.

Lastly, while carbon-based nanomaterials hold immense promise for revolutionizing healthcare, their successful integration into clinical practice requires concerted efforts from multidisciplinary teams. Addressing safety concerns, optimizing synthesis methods, obtaining regulatory approvals, and ensuring ethical considerations are paramount for unlocking the full clinical benefits of CBNs. With continued research and collaboration, CBN-based healthcare technologies have the potential to significantly improve diagnostics, therapies, and overall patient outcomes, ushering in a new era of advanced healthcare solutions.
